# Identifying individual hospital levels of maternal care using administrative data

**DOI:** 10.1186/s12913-021-06516-y

**Published:** 2021-06-02

**Authors:** Sara C. Handley, Molly Passarella, Sindhu K. Srinivas, Scott A. Lorch

**Affiliations:** 1 Division of Neonatology, Department of Pediatrics, The Children’s Hospital of Philadelphia and the Perelman School of Medicine-University of Pennsylvania, Philadelphia, PA USA; 2 Leonard Davis Institute of Health Economics, University of Pennsylvania, Philadelphia, PA USA; 3 The Maternal and Child Health Research Center, Department of Obstetrics and Gynecology and the Perelman School of Medicine-University of Pennsylvania, Philadelphia, PA USA

**Keywords:** Perinatal care, Regionalization, Risk-appropriate

## Abstract

**Background:**

The goal of regionalized perinatal care, specifically levels of maternal care, is to improve maternal outcomes through risk-appropriate obstetric care. Studies of levels of maternal care are limited by current approaches to identify a hospital’s level of care, often relying on hospital self-reported data, which is expensive and challenging to collect and validate. The study objective was to develop an empiric approach to determine a hospital’s level of maternal care using administrative data reflective of the patient care provided and apply this approach to describe the levels of maternal care available over time.

**Methods:**

Retrospective cohort study of mother-infant dyads who delivered in California, Missouri, and Pennsylvania hospitals from 2000 to 2009. Linked mother-infant administrative records with an infant born at 24–44 weeks’ gestation and a birth weight of 400–8000 g were included. Using the American College of Obstetricians and Gynecologists and the Society for Maternal Fetal Medicine descriptions of levels of maternal care, four levels were classified based on the appropriate location of care for patients with specific medical or pregnancy conditions. Individual hospitals were assigned a level of maternal care annually based on the volume of patients who delivered reflective of the four classified levels as determined by International Classification of Diseases and Current Procedural Terminology.

**Results:**

Based on the included 6,895,000 mother-infant dyads, the obstetric hospital levels of maternal care I, II, III and IV were identified. High-risk patients more frequently delivered in hospitals with higher level maternal care, accounting for 8.9, 10.9, 13.8, and 16.9% of deliveries in level I, II, III and IV hospitals, respectively. The total number of obstetric hospitals decreased over the study period, while the proportion of hospitals with high-level (level III or IV) maternal care increased. High-level hospitals were located in more densely populated areas.

**Conclusion:**

Identification of the level of maternal care, independent of hospital self-reported variables, is feasible using administrative data. This empiric approach, which accounts for changes in hospitals over time, is a valuable framework for perinatal researchers and other stakeholders to inexpensively identify measurable benefits of levels of maternal care and characterize where specific patient populations receive care.

**Supplementary Information:**

The online version contains supplementary material available at 10.1186/s12913-021-06516-y.

## Introduction

One approach to address issues in maternal health, such as the rising maternal mortality rates [1] and burden of severe maternal morbidity [2], is to improve the regionalization of perinatal care. The goal of regionalization is “to improve patient outcomes by directing patients to facilities with optimal capabilities for a given type of illness or injury” [3]. Hospital levels of care, a fundamental aspect of regionalization, describe the types of hospital-based services available to patients, which in turn describes the types of patients who can be adequately cared for at a certain hospital. Motivated by the American Academy of Pediatrics (AAP) levels of neonatal care, which have been associated with improved neonatal outcomes [4–10], levels of maternal care were first published in the United States (US) by the American College of Obstetricians and Gynecologists (ACOG) and the Society for Maternal-Fetal Medicine (SMFM) in 2015 and updated in 2019 [11, 12]. The ACOG/SMFM statement describes level I care as basic care, level II as specialty care, level III as subspecialty care, and level IV as care provided in Regional Perinatal Health Care Centers [11, 12].

However, the level of maternal care provided at individual hospitals is currently not easily accessible or publicly available. While some states in the US have active levels of maternal care verification programs, these programs are resource intensive requiring significant time and financial support. Most research studies depend on hospital self-reported measures, such as those from research surveys, the American Hospital Association (AHA) annual survey, or the Centers for Disease Control and Prevention’s Levels of Care Assessment Tool (CDC LOCATe), to determine the available level of maternal care [13–16]. These self-report measures, though, are often overstated by hospitals [17]. Thus, the level of care based on the characteristics of patients who actually received care in a given hospital may more accurately reflect the level of care available. To advance perinatal regionalization research, knowledge of associated patient outcomes, and optimize risk-appropriate care delivery, alternate approaches to ascertain the level of maternal care provided at hospitals is needed.

The objective of this study was to develop an empiric approach to determine the level of maternal care available at a hospital using administrative data reflective of the patient care provided. The secondary objective was to apply this approach to describe available levels of maternal care over time. To achieve these objectives, we leveraged a cohort of almost seven million linked mother-infant administrative records reflecting all births in California, Missouri, and Pennsylvania between 2000 and 2009. This study provides an alternate framework for level of care determination, new insights into how available levels of maternal care change over time, and data to inform future research regarding levels of maternal care.

## Materials and methods

### Study design

This is a retrospective, population-based cohort study of hospital-based deliveries in California, Missouri, and Pennsylvania from January 1, 2000 to December 31, 2009 using linked state-level hospital administrative data, birth certificates, and death certificates. California, Missouri, and Pennsylvania were chosen given the availability of valid, accessible, mother-infant linked data, representing diverse populations, and different perinatal care systems. The state-level hospital administrative data includes data on all hospital discharges including International Classification of Diseases (ICD) and Current Procedural Terminology (CPT) codes associated with the hospitalization. Birth and death certificates contain validated variables including birth weight, gestational age, mode of delivery [18], race/ethnicity [19], and insurance [20].

Linkage of mother-infant data was performed by the individual states prior to data distribution. Using previously published methods, the mother-infant match rate was 94% [21]. Of the unmatched records, more than 80% of the unmatched birth certificates were missing a hospital identifier, suggesting delivery at home or in a birthing center, and the remainder were unmatched secondary to coding errors. Unmatched records had gestational age and racial/ethnic distributions similar to the matched records. Unmatched records were excluded from the study. Infant records were included if the infant’s gestational age was between 24 and 44 weeks and the birthweight was between 400 and 8000 g and did not exceed five standard deviations from the mean for gestational age. Records with an incorrect or abnormal hospital identifier were also excluded. This study was deemed exempt by the Institutional Review Board at the Children’s Hospital of Philadelphia, as it did not meet the criteria for human subjects research.

### Study variables

Based on the AGOG/SMFM description of levels of maternal care (Table 1), relevant study variables were identified (Table 2). Univariate analyses of patient characteristics included maternal demographics, maternal comorbid and pregnancy associated conditions, and neonatal characteristics. The complete list of associated ICD-9 and CPT codes are included in the Additional file 1, ICD-9 and CPT Codes for Study Variables.

**Table 1:** Characteristics of Levels of Maternal Care and Associated Thresholds for Empiric Levels of Maternal Care

Maternal level	Capabilities^**a**^	Health care providers^**a**^	Examples of appropriate patients^**b**^	Threshold of ‘high risk patients’/year^**c**^
Level I (Basic Care)	-Capability/equipment to provide low-risk and appropriate moderate-risk maternal care and readiness at all times to initiate emergency procedures -Stabilization and the ability to facilitate transport to a higher-level hospital when necessary -Collaboration with higher-level facility partners, to initiate/sustain education and QI programs to maximize patient safety	-Every birth attended by a midwife, family physician or ob-gyn and an appropriately trained/qualified RN -Physician with privileges to perform emergency cesarean delivery readily available at all times -Primary maternal care providers, including midwives, family physicians, or ob-gyns readily available at all times -Appropriately trained/qualified RNs with level-appropriate competencies readily available at all times -RN leadership has level-appropriate training and experience in maternal care -Anesthesia providers for labor and surgical anesthesia readily available at all times	-Term twin gestation -Trial of labor after cesarean delivery -Uncomplicated cesarean delivery -Preeclampsia without severe features at term	
Level II (Specialty Care)	Level I facility capabilities plus -CT scan, MRI, non-obstetric US imaging, and maternal echocardiography with interpretation readily available daily -Standard obstetric US imaging with interpretation readily available at all times	Level I facility health care providers plus -Ob-gyn readily available at all times -Physician OB leadership board-certified in ob-gyn with experience in obstetric care -MFM readily available at all times for consultation onsite, by phone, or by telemedicine -Anesthesiologist readily available at all times -Internal or family medicine physicians and general surgeons readily available at all times for obstetric patients	-Severe preeclampsia -Placenta previa with no prior uterine surgery	≥5/year Preterm (< 37 week) multiples ≥3/year Primary Cesarean section for placenta previa ≥4/year Severe hypertension after 34 weeks’ gestation
Level III (Subspecialty Care)	Level II facility capabilities plus -All blood components available in-house -CT scan, MRI, maternal echocardiography, and non-obstetric US imaging services and interpretation readily available at all times -Specialized obstetric US and fetal assessment with interpretation readily available at all times -Interventional radiology (capable of uterine artery embolization) readily available at all times -Equipment/personnel physically present at all times to ventilate/monitor women until ICU transfer -Onsite medical/surgical ICUs that accept pregnant and postpartum women. ICUs have adult critical care providers physically present at all times -MFM readily available at all times to communicate/consult for all obstetric ICU patients -Mechanism to facilitate/accept maternal transfers/transports -Provide outreach education and patient transfer feedback to level I and II centers -Provide Perinatal system leadership if acting as a regional center (see Level IV)	Level II facility health care providers plus -Nursing leaders and adequate number of RNs who have training and experience in the management of women with complex and critical maternal illnesses and obstetric complications. -Board certified ob-gyn physically present at all times. -An MFM with inpatient privileges readily available at all times, either onsite, by phone, or by telemedicine. MFM must be able to be onsite and provide direct care within 24 h. -Director of MFM service is a board-certified MFM. -Director of obstetric anesthesia services is a board-certified anesthesiologist with OB anesthesia fellowship training or experience in OB anesthesia. -Full complement of subspecialists, such as critical care, general surgery, infectious diseases, hematology, cardiology, nephrology, neurology, gastroenterology, internal medicine, behavioral health, and neonatology readily available for inpatient consultation at all times	-Suspected placenta accreta or previa with prior uterine surgery -Suspected placenta percreta -Adult respiratory syndrome -Expectant management of early severe preeclampsia at less than 34 weeks of gestation	≥3/year Previous Cesarean section and a placenta previa ≥5/year Preeclampsia before 34 weeks’ gestation ≥3/year Severe hypertension or Eclampsia before 34 weeks’ gestation ≥3/year Acute Respiratory Distress Syndrome
Level IV (Regional Perinatal Health Care Centers)	Level III facility capabilities plus -On-site medical/surgical care for complex maternal conditions with available ICU beds -On-site ICU care for obstetric patients with primary or co-management by MFM If the woman must be transported by ambulance to the ICU, this is not considered onsite. -Perinatal system leadership, including facilitation of collaboration with facilities in the region, analysis and review of system perinatal outcome and quality data, provision of outreach education and assistance with QI	Level III health care providers plus -MFM team with expertise in highly complex, critically ill, or unstable maternal patients -Board-certified MFM with full inpatient privileges readily available at all times, including co-management of ICU-admitted obstetric patients -Nursing Service Line leadership with advanced degree and national certification - RNs with experience in complex medical illnesses/obstetric complications and collaboration between critical care and obstetric RNs -Board-certified anesthesiologist with OB anesthesia fellowship training or experience in OB anesthesia physically present at all times -At least one of the following subspecialties readily available at all times onsite: neurosurgery, cardiac surgery, or transplant. If all three subspecialties are not available, there should be a process to transfer women to a facility with the service	-Severe maternal cardiac conditions -Severe pulmonary hypertension or liver failure -Pregnant women requiring neurosurgery or cardiac surgery -Pregnant women in unstable condition and in need of an organ transplant	≥3/year Severe chronic medical conditions^d^ ≥5/year Severe cardiac conditions^e^

Abbreviations: *OB* obstetric, *RN* nurse, *CT* computerized tomography, *MRI* magnetic resonance imaging, *US* ultrasound, *MFM* maternal fetal medicine, *ICU* intensive care unit

^a^Text adapted from 2019 Levels of Maternal Care Statement: Obstetric Care Consensus No. 9: Levels of Maternal Care Obs Gynecol. 2019;134 (2):e41-e55)

^b^Text adapted from 2015 Levels of Maternal Care Statement: Obstetric Care Consensus No. 2: Levels of maternal care. Obs Gynecol. 2015;125 (2):502–515)

^c^The study determined annual threshold was created by the authors considering both the volume of patients to clinically indicate a hospital’s intent to deliver the different types of appropriate patients and calculated the sensitivity, specificity, and Youden’s index, summary measure of the Receiver Operative Characteristic curve, for each type of high-risk case to inform threshold cut points

^d^Severe chronic medical conditions: pulmonary hypertension, liver failure, dialysis, and organ transplant

^e^Severe cardiac conditions: chronic heart disease, hypertrophic cardiomyopathy, acute/subacute endocarditis, constrictive pericarditis, tamponade, complete atrioventricular block, cardiac device in situ, atrial fibrillation, atrial flutter, congestive heart failure, mitral stenosis, atrial stenosis, dual valve disease, value replacement

**Table 2:** Maternal and Infant Characteristics Delivering in Non-Obstetric and Obstetric Hospitals by Empiric Maternal Level of Care in California, Missouri, and Pennsylvania, 2000–2009

Variable	Non-Obstetric *N* = 10,859 (0.2%)	Level I *N* = 467,368 (6.8%)	Level II *N* = 2,861,713 (41.5%)	Level III *N* = 2,442,605 (35.4%)	Level IV *N* = 1,113,055 (16.1%)	*P*-value
State						< 0.001
CA	4141 (0.1%)	178,951 (3.6%)	2,079,001 (41.8%)	2,055,616 (41.3%)	661,168 (13.3%)	
MO	4484 (0.6%)	87,528 (12.1%)	268,344 (37.0%)	106,355 (14.7%)	257,647 (35.6%)	
PA	2234 (0.2%)	200,889 (16.9%)	514,368 (43.1%)	280,634 (23.5%)	194,240 (16.3%)	
Deliveries/year (median, IQR)	74 (59–87)	443 (298–570)	1562 (1099–2091)	3318 (2648–4041)	4511 (3035–6644)	< 0.001
**Maternal characteristics, N (%)** (unless otherwise noted)						
Maternal age (years; median, IQR)	25 (21–30)	26 (22–31)	27 (23–32)	28 (24–33)	28 (23–33)	< 0.001
Race						< 0.001
White, Non-Hispanic	8943 (82.4%)	332,532 (71.2%)	1,306,403 (45.7%)	935,697 (38.3%)	492,285 (44.2%)	
Black, Non-Hispanic	289 (2.7%)	20,293 (4.3%)	182,813 (6.4%)	170,257 (7.0%)	158,016 (14.2%)	
Hispanic	1025 (9.4%)	85,636 (18.3%)	1,062,409 (37.1%)	975,757 (40.0%)	315,668 (28.4%)	
Asian/Pacific Islander	195 (1.8%)	13,680 (2.9%)	249,544 (8.7%)	312,415 (12.8%)	118,043 (10.6%)	
Other	407 (3.8%)	15,227 (3.3%)	60,544 (2.1%)	48,479 (2.0%)	29,043 (2.6%)	
Insurance						< 0.001
FFS	2184 (20.1%)	75,691 (16.2%)	188,818 (6.6%)	120,363 (4.9%)	56,676 (5.1%)	
HMO	1690 (15.6%)	134,753 (28.8%)	1,298,549 (45.4%)	1,270,782 (52.0%)	527,953 (47.4%)	
Public	5864 (54.0%)	227,233 (48.6%)	1,264,669 (44.2%)	981,462 (40.2%)	493,018 (44.3%)	
Other	1121 (10.3%)	29,691 (6.4%)	109,677 (3.8%)	69,998 (2.9%)	35,408 (3.2%)	
Education						< 0.001
No High School	520 (4.8%)	30,481 (6.5%)	256,725 (9.0%)	197,890 (8.1%)	69,522 (6.3%)	
Some High School	2217 (20.4%)	82,707 (17.7%)	490,139 (17.1%)	393,694 (16.1%)	172,056 (15.5%)	
High School Diploma/GED	4364 (40.2%)	162,316 (34.7%)	812,643 (28.4%)	608,347 (24.9%)	276,107 (24.8%)	
At least Some College	3636 (33.5%)	186,899 (40.0%)	1,260,098 (44.0%)	1,180,600 (48.3%)	563,492 (50.6%)	
Missing	122 (1.1%)	4965 (1.1%)	42,108 (1.5%)	62,074 (2.5%)	31,878 (2.9%)	
**Maternal comorbid and pregnancy associated conditions, N (%)**						
Chronic hypertension	66 (0.6%)	3100 (0.7%)	19,924 (0.7%)	21,238 (0.9%)	15,717 (1.4%)	< 0.001
PIH	356 (3.3%)	12,369 (2.7%)	79,791 (2.8%)	83,689 (3.4%)	58,081 (5.2%)	< 0.001
Severe PIH/Eclampsia	71 (0.7%)	2369 (0.5%)	22,327 (0.8%)	27,832 (1.1%)	19,506 (1.8%)	< 0.001
Gestational diabetes	359 (3.3%)	17,911 (3.8%)	138,214 (4.8%)	145,951 (6.0%)	70,231 (6.3%)	< 0.001
Diabetes mellitus	35 (0.3%)	2236 (0.5%)	17,287 (0.6%)	20,877 (0.9%)	13,156 (1.2%)	< 0.001
Renal disease	27 (0.3%)	723 (0.2%)	3514 (0.1%)	3006 (0.1%)	2251 (0.2%)	< 0.001
Dialysis	0 (0.0%)	5 (0.0%)	99 (0.0%)	149 (0.01%)	134 (0.01%)	< 0.001
Organ transplant	0 (0.0%)	21 (0.0%)	140 (0.0%)	253 (0.01%)	368 (0.03%)	< 0.001
Severe chronic condition^a^	2 (0.02%)	61 (0.01%)	660 (0.02%)	954 (0.04%)	1085 (0.1%)	< 0.001
Severe cardiac condition^b^	9 (0.1%)	376 (0.08%)	2856 (0.1%)	3515 (0.1%)	3346 (0.3%)	< 0.001
Placenta previa	47 (0.4%)	1537 (0.3%)	14,011 (0.5%)	16,393 (0.7%)	7767 (0.7%)	< 0.001
Placenta previa after CS	10 (0.1%)	286 (0.1%)	2859 (0.1%)	3935 (0.2%)	1933 (0.2%)	< 0.001
Multiple gestation	132 (1.2%)	5704 (1.2%)	61,439 (2.2%)	78,841 (3.2%)	43,408 (3.9%)	< 0.001
High-risk patients^c^	982 (9.0%)	41,527 (8.9%)	311,689 (10.9%)	337,059 (13.8%)	188,125 (16.9%)	< 0.001
Cesarean section	3150 (29.0%)	126,928 (27.2%)	819,661 (28.6%)	757,811 (31.0%)	340,778 (30.6%)	< 0.001
**Neonatal characteristics, N (%)** (unless otherwise noted)						
Male	5534 (51.0%)	238,891 (51.1%)	1,463,488 (51.1%)	1,252,240 (51.3%)	570,285 (51.2%)	< 0.001
Birthweight	3317 (3000,3629)	3374 (3061,3686)	3371 (3041,3685)	3345 (3005,3660)	3319 (2965,3657)	< 0.001
GA, weeks	39 (38,40)	39 (38,40)	39 (38,40)	39 (38,40.)	39 (38,40)	< 0.001
GA categories, weeks						< 0.001
GA < 28 weeks	42 (0.4%)	799 (0.2%)	8510 (0.3%)	13,045 (0.5%)	9632 (0.9%)	
GA 28–31 weeks	79 (0.7%)	1682 (0.4%)	19,247 (0.7%)	29,233 (1.2%)	19,549 (1.8%)	
GA 32–36 weeks	767 (7.1%)	29,030 (6.2%)	229,616 (8.0%)	236,880 (9.7%)	122,070 (11.0%)	
GA 37–41 weeks	9607 (88.5%)	420,510 (90.0%)	2,473,357 (86.4%)	2,046,481 (83.8%)	921,954 (82.8%)	
GA > 41	364 (3.4%)	15,347 (3.3%)	130,983 (4.6%)	116,966 (4.8%)	39,850 (3.6%)	

Abbreviations: *CA* California, *MO* Missouri, *PA* Pennsylvania, *IQR* interquartile range, *FFS* fee for service, *HMO* health maintenance organization, *GED* general education diploma, *PIH* pregnancy induced hypertension, *CS* Cesarean section, *GA* gestational age

^a^Severe chronic medical conditions: pulmonary hypertension, liver failure, dialysis, and organ transplant

^b^Severe cardiac conditions: chronic heart disease, hypertrophic cardiomyopathy, acute/subacute endocarditis, constrictive pericarditis, tamponade, complete atrioventricular block, cardiac device in situ, atrial fibrillation, atrial flutter, congestive heart failure, mitral stenosis, atrial stenosis, dual valve disease, value replacement

^c^High-risk patients have any of the listed maternal comorbid and pregnancy associated conditions

### Level of maternal care

The ACOG/SMFM statement describes levels of maternal care as basic care (level I), specialty care (level II), subspecialty care (level III), and Regional Perinatal Health Care Centers (level IV) (Table 1). The statement provides the associated definition, capabilities, and health care providers for each level of maternal care [11, 12]. In the 2015 statement, examples of appropriate patients for each level of maternal care were also provided [11]. We used the information in the ACOG/SMFM statements, specifically the examples of appropriate patients which should reflect hospital capabilities and health care providers, to empirically classify hospitals into one of the four levels of maternal care based on the types of patients that a hospital with that level should have treated in that year. Empiric levels of maternal care were solely based on patient data and did not include direct measures of hospital capabilities or health care providers. We considered hospitals with < 100 deliveries/year non-obstetric hospitals.

We used the aforementioned ACOG/SMFM examples of appropriate patients and state-level hospital administrative data, ICD-9 and CPT codes, to assign hospitals an empiric level of maternal care. We assigned an empiric level of maternal care to each hospital based on a threshold number of appropriate patients per year (Table 1). We determined the threshold of appropriate ‘high-risk’ patients for each level of maternal care by first examining Youden’s index, a summary measure of the Receiver Operating Characteristic curve that supports selection of optimal threshold values, the sensitivity, and specificity for each type of high-risk patient (Supplemental Table 1) [22]. Then, given the potential for an urgent, emergent, or unexpected delivery at a low-level hospital of a patient whose condition may warrant a higher level of maternal care, we examined the face validity of the Youden-based cutpoints while considering the volume of patients indicating a hospital’s intent to deliver different types of appropriate patients and clinically does not differ between states (Supplemental Table 1). For example, to differentiate between level II and III hospitals, the optimal cutpoint per Youden’s index for the number of patients with severe hypertension or eclampsia before 34 weeks’ gestation was 2 (sensitivity 95%, specificity 96%, area under ROC curve 0.95) and directly informed our threshold of ≥3/year. For previous cesarean section and a placenta previa, the optimal cutpoint was 1, which was deemed clinically inappropriate as it is plausible that 0–2 patients/year may present to a level II hospital and require urgent delivery, thus we identified an alternate threshold (≥3/year) to maximize correct classification. Hospitals had to meet 2 of 3 high-risk patient thresholds to be categorized as a level II, 3 of 4 high-risk patient thresholds to be categorized as a level III, and both high-risk patient thresholds to be categorized as a level IV (Table 1). Hospitals had to meet more than one threshold criteria because levels of maternal care describe hospitals with the ability to care for more than one type of high-risk patient while meeting all criteria for level II or III was thought to be too stringent and could increase misclassification. Potential inconsistencies (e.g., hospitals with varying levels of maternal care each year) in the assignment of a hospital’s empiric level of maternal care were discussed amongst authors until agreement was reached. Levels were assigned annually to account for changes in hospital services and obstetric unit closures [11, 12]. We examined the distribution of high-risk patients delivering across our empiric levels of maternal care as a basic validation assessment [23]. Verification of individual hospitals level of maternal care are not yet publicly available in the states studied.

### Data analysis

Determination of the empiric level of maternal care was conducted by year and state. Descriptive analyses were completed for the cohort and stratified by state secondary to differences in perinatal health systems. The number of non-obstetric hospitals and obstetric hospitals with level I, II, III, and IV maternal care were calculated. Stata version 15 (StataCorp, College Station, TX) was used for the univariate analyses, which compared patient characteristics of non-obstetric hospitals and the four levels of maternal care using Pearson Chi-Squared and Kruskal-Wallis tests. The hospital location geocodes were used to construct maps overlying the population density using ArcGIS (Esri, Redlands, CA).

## Results

**Fig. 1:**
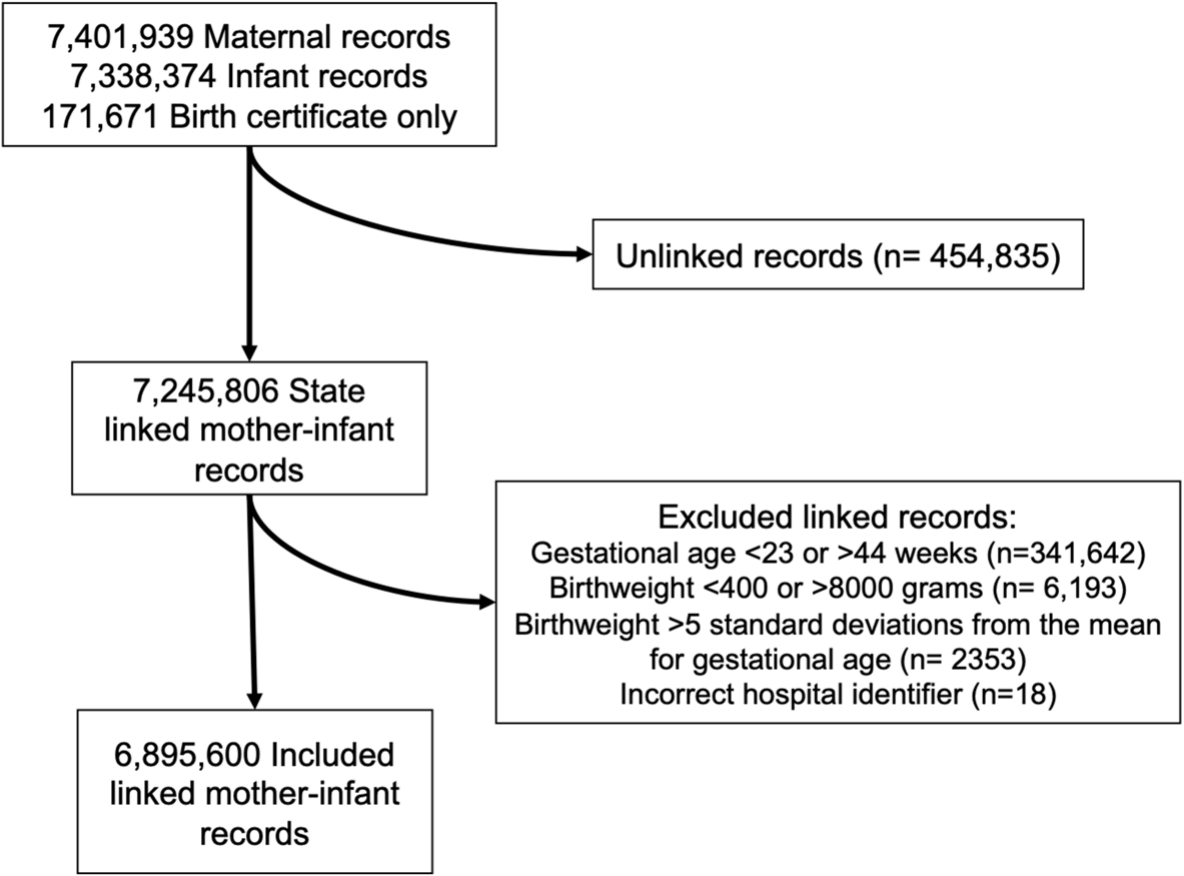
Cohort Identification Flow Diagram

The analytic cohort included 6,895,600 mother-infant dyads (Fig. 1) who delivered at more than 500 hospitals over 10 years. The characteristics of patients delivering in non-obstetric and obstetric hospitals of varying levels of maternal care differed (Table 2). Non-obstetric hospitals cared for more patients with public insurance, whereas, high-risk patients such as those of higher maternal age and with those with comorbid and pregnancy associated conditions delivered at hospitals with a higher level of maternal care, as shown by the Table columns with the percentage of patients with each condition by level of care. When stratifying by state, the increased proportion of high-risk patients delivering in higher level hospitals persisted (Tables 2, 3 and 4 in Supplemental materials). Additionally, there was an increased percentage non-Hispanic Blacks and Asian/Pacific Islanders in hospitals with higher level maternal care, reflecting the co-located nature of these populations in the states studied and high level centers in metropolitan areas.

**Fig. 2:**
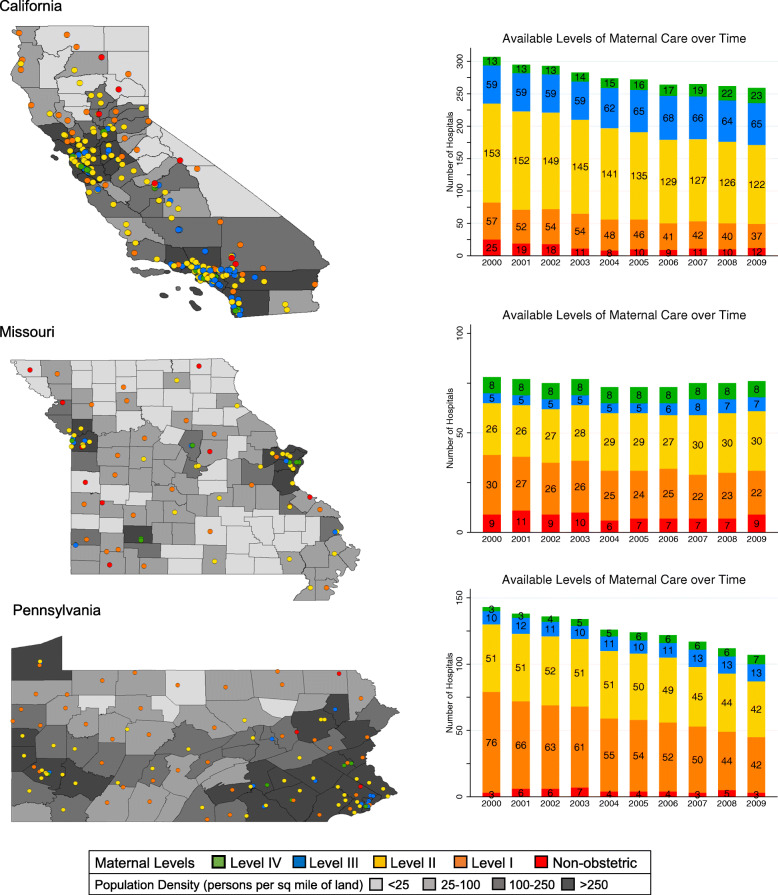
Distribution of Non-obstetric and Obstetric Hospitals and Available Empiric Levels of Maternal Care. Geographic distribution and associated number of non-obstetric and obstetric hospitals by level of maternal care in California, Missouri, and Pennsylvania from 2000 to 2009 with maps illustrating the distribution of institutions in 2009

The total number of hospitals, both obstetric and non-obstetric, declined during the study period. In 2000, 491 of 528 hospitals were identified as obstetric hospitals, whereas at the end of the study period, 2009, 418 of 442 hospitals were identified as obstetric hospitals. Similarly, in 2000, 37 of 528 hospitals were non-obstetric and by 2009, 24 of 442 hospitals were non-obstetric. The decrease in obstetric and non-obstetric hospitals resulted in an increase of births in obstetric hospitals from 93 to 94.6%. Maps of each state illustrate the distribution of non-obstetric and obstetric hospitals by their empiric level of maternal care in 2009 (Fig. 2). Hospitals with higher levels of maternal care and in close proximity to one another were consistently in urban areas with higher population density. The number of obstetric hospitals decreased in all states over the study period, by 3% in Missouri, 12% in California, and 26% in Pennsylvania, primarily from reductions in the number of hospitals providing level I and II care (Fig. 2).

Based on the empiric levels of maternal care, the majority of women delivered at hospitals with level II care, 41.8, 37.0, and 43.1% in California, Missouri, and Pennsylvania, respectively (Table 2). The proportion of deliveries at a non-obstetric hospital in Missouri (0.9%) was almost five times higher than Pennsylvania (0.2%) and 10 times higher than California (0.08%). The proportion of deliveries in hospitals with level I maternal care varied widely, from 3.6% in California to 12.1% in Missouri and 16.9% in Pennsylvania. The frequency of women in Missouri (35.6%) delivering at a hospital with level IV maternal care was nearly double that of Pennsylvania (16.3%) and California (13.3%).

## Discussion

In this study, we developed an empiric approach to determine levels of maternal care at individual hospitals in California, Missouri, and Pennsylvania using administrative data. Applying this approach, high-risk patients more frequently delivered at hospitals with higher levels of maternal care, which is consistent with risk-appropriate care delivery. During the study period the number of obstetric hospitals decreased by nearly 15%. As expected, hospitals with higher levels of maternal care were in metropolitan centers. To our knowledge, this is the first study to describe an empiric approach to determine the available level of maternal care using administrative data reflective of the patient care provided, independent of self-reported hospital services and subspecialist availability.

Identifying levels of maternal care using only administrative data has not been described in the literature, however using ICD codes to identify levels of maternal care may be preferable to other methods. First, as more recent data become available and ICD codes are revised, variable crosswalks (i.e. ICD-9 to ICD-10) will make the continued application of this approach feasible. Second, ICD codes determine diagnosis-related group (DRG) codes which determine payment, thus the ICD codes associated with patients should reflect conditions that warrant billing, increasing reliability. Third, hospitals unreliably self-assess their capabilities, with 18% of neonatal units overestimating and 50% obstetric units incorrectly reporting their level of care in prior audits [17, 24]. Fourth, our approach accounts for the dynamic nature of maternal care delivery as we determine the level of care annually and the empirically derived levels reflect the care actually being provided increasing its relevance. Fifth, in the US there is limited oversight regarding where pregnant patients deliver and although maternal risk assessment during the antenatal period or at the time of delivery may identify high-risk patients and clinicians may recommend delivery in a hospital with higher level care, ultimately the location of maternal care often reflects personal choice and is influenced by free market economics. Finally, an analogous empiric methodologic approach has been utilized in studies of neonatal levels of care, associated with outcomes, and used to inform neonatal regionalization efforts supporting the validity of this approach [9, 10].

Previous studies of levels of maternal care are currently limited by a lack of national level reports and reliance on self-reported survey measures to determine the level of care. The prior literature, though, supports the need to accurately identify the level of maternity care provided by hospitals. Consistent with our findings, two cross-sectional studies by Srinivas et al. and Easter et al. reflective of nine and seven states respectively, reported high risk patients are more likely to be managed in high level hospitals [13, 14]. Easter et al. also reported 2.4% of patients delivered in a hospital with an inappropriate level of maternal care, though it is unclear if these deliveries were emergent or in a hospital with a hospital-perceived higher level of care [14]. To date, data regarding maternal levels of care and outcomes are mixed as Srinivas et al. found women with cardiac conditions had a significantly lower odds or mortality when delivering in a level I compared to a level IV hospital [13] while Vanderlaan et al. found no difference in maternal outcomes by level of maternal care in Georgia [15]. The variation we report in levels of maternal care between states and nearly 15% decrease in the total number of obstetric hospitals, is also consistent with previous literature [25]. The decrease in available obstetric services after obstetric hospital closures has been associated with increased travel distance to an obstetric hospital, increased delivery outside of the hospital or delivery at a hospital without obstetric services, and increased perinatal and neonatal mortality [26–28], outcomes highlighting the potential need for perinatal regionalization.

Our methodology offers researchers and leaders in perinatal care delivery and policy another approach to more systematically and widely identifying levels of maternal care. Compared to surveys or site visits, this empiric approach is much less resource intensive and more accessible. Importantly, this ICD code based approach, allows for international applications, as ICD codes are used in over 100 countries worldwide [29]. Studying the empiric levels of maternal care provided across countries may illuminate how and where obstetric care is being delivered and facilitate more meaningful international comparisons. As new data are available, applying this approach to other epochs, states, and countries should be pursued. This research may expedite further studies of levels of maternal care leading to a better understanding the measurable benefits of levels of maternal care and allowing for the characterization of specific patient populations who may benefit from a particular level of maternal care.

### Strengths and limitations

One of the primary limitations of this approach is the inability to validate the empiric level of maternal care assigned to each hospital with an external data source. Unlike the level of neonatal or trauma care available at a hospital, which have been the focus of national surveys, inventories, and verification programs, such data are not yet available for maternal care [30–32]. This potential approach is also complicated by issues obtaining linked mother-infant records and survey data with hospitals over-reporting their capabilities [17]. As described by Easter el al, the delivery of high-risk patients at hospitals with low level maternal care may have influenced the level of maternal care assignments [14]. Our approach acknowledges this possibility and thus the classifications of the four levels of maternal care each incorporate multiple types of high-risk patients. Another limitation is that non-hospital births, such as those occurring at home or in non-hospital affiliated birth centers, are not included in this dataset. Indications for delivery in the non-hospital setting or a specific level of maternal care cannot be determined. This methodology does not incorporate information on hospital capabilities and providers, though to some extent the care provided to patients should reflect available hospital services and providers. Strengths of our study include that it describes the actual level of care provided to patients, which is important to consider when assessing associations with outcomes. Additionally, we assigned the level of maternal care independent of self-reported measures. This approach is significantly less expensive and provides level of maternal care data much more quickly than and onsite survey approach. Finally, the use of ICD codes allows for application of the approach outside of the US.

## Conclusions

This study describes an approach to identify and assign hospital levels of maternal care. Additionally, this study highlights the evolving landscape of available obstetric services and provides new opportunities to study levels of maternal care. Given the current rates of maternal morbidity and mortality in the US and intent for levels of maternal care to improve perinatal care, a systematic, widely available, efficient approach to identifying levels of maternal care and study associations is crucial to optimize and improve perinatal outcomes.

## Supplementary Information

**Additional file 1.** ICD-9 and CPT Codes for Study Variables.

**Additional file 2: Supplemental Table 1**. Sensitivity and Specificity of High-Risk Patient Thresholds to Identify Maternal Levels of Care. **Supplemental Table 2**. Maternal and Infant Characteristics Delivering in Non-Obstetric and Obstetric Hospitals by Empiric Maternal Level of Care in California, 2000–2009. **Supplemental Table 3**. Maternal and Infant Characteristics Delivering in Non-Obstetric and Obstetric Hospitals by Empiric Maternal Level of Care in Missouri, 2000–2009. **Supplemental Table 4**. Maternal and Infant Characteristics Delivering in Non-Obstetric and Obstetric Hospitals by Empiric Maternal Level of Care in Pennsylvania, 2000–2009.

## Data Availability

The data that support the findings of this study are available from the individual states but restrictions apply to the availability of these data, which were used under data use agreements between each state department of health, vital statistics, and The Children’s Hospital of Philadelphia for the current study, and so are not publicly available. Data are however available from the authors upon reasonable request and with permission of the respective state agencies.

## Abbreviations

AAP: American Academy of Pediatrics
US: United States
ACOG: American College of Obstetricians and Gynecologists
SMFM: Society for Maternal-Fetal Medicine
AHA: American Hospital Association
CDC LOCATe: Centers for Disease Control and Prevention’s Levels of Care Assessment Tool
ICD: International Classification of Diseases
CPT: Current Procedural Terminology
