# Supplementary material for: Identifying individual hospital levels of maternal care using administrative data
Source: BMC Health Serv Res. 2021 Jun 2;21:538. doi: 10.1186/s12913-021-06516-y (PMC8171026; doi:10.1186/s12913-021-06516-y)
Supplement: Supplementary file 1 — Additional file 1. ICD-9 and CPT Codes for Study Variables. [file 12913_2021_6516_MOESM1_ESM.docx]

**Title**: Identifying Individual Hospital Levels of Maternal Care using Administrative Data

**Authors**: Sara C. Handley MD, MSCE^1,2^, Molly Passarella MS^1^, Sindhu K. Srinivas, MD, MSCE^2,3^, Scott A. Lorch MD MSCE^1,2^

**Institutions**: ^1^Division of Neonatology, Department of Pediatrics, The Children’s Hospital of Philadelphia and the Perelman School of Medicine-University of Pennsylvania, Philadelphia, PA; ^2^Leonard Davis Institute of Health Economics, University of Pennsylvania, Philadelphia, PA, ^3^The Maternal and Child Health Research Center, Department of Obstetrics and Gynecology, Perelman School of Medicine-University of Pennsylvania, Philadelphia, PA

Appendix 1. ICD-9 and CPT Codes for Study Variables

| Maternal comorbid conditions | |
| --- | --- |
| Chronic hypertension | 642.00, 642.01, 642.02, 642.03, 642.04, 642.10, 642.11, 642.12, 642.13, 642.14, 642.20, 642.21, 642.22, 642.23, 642.24 |
| Diabetes mellitus | 648.0, 648.00, 64801, 648.02, 648.03, 648.04, 250, 250.0, 250.00, 250.01, 250.02, 250.03, 250.1, 250.10, 250.11, 250.12, 250.13, 250.2, 250.20, 250.21, 250.22, 250.23, 250.3, 250.30, 250.31, 250.32, 250.33, 250.4, 250.40, 250.41, 250.42, 250.43, 250.5, 250.50, 250.51, 250.52, 250.53, 250.6, 250.60, 250.61, 250.62, 250.63, 250.7, 250.70, 250.71, 250.72, 250.73, 250.8, 250.80, 250.81, 250.82, 250.83, 250.9, 250.90, 250.91, 250.92, 250.93, 357.2, 360.2, 362.01, 366.41 |
| Renal disease | 646.2, 646.20, 646.21, 646.22, 646.23, 646.24 |
| Dialysis | V45.1, V45.11, V45.12, V56, V56.0, V56.1, V56.2, V56.3, V56.8, 399.5, 549.8 |
| Organ transplant | V42.0, V42.1, V42.2, V42.3, V42.4, V42.5, V42.6, V42.7, V42.81, V42.82, V42.83, V42.84, V42.89, V42.9, 996.80, 996.81, 996.82, 996.83, 996.84, 996.85, 996.86, 99687, 996.88, 996.89 |
| Severe chronic medical conditions  (pulmonary hypertension, liver failure, dialysis, organ transplant) | 416.0, 416.8, V45.1, V45.11, V45.12, V56, V56.0, V56.1, V56.2, V56.3, V56.8, 996.56, 996.68, V56.2, V56.31, V56.32, V56.8, 45.821, 57.1, 57.11, 57.12, 57.13, 57.14, 57.15, 57.16, 57.18, 57.19, 070.22, 070.23, 070.32, 070.33, 070.44, 070.54, 456.0,456.1, 456.2, 456.20, 456.21, 571, 571.0, 571.1, 571.2, 571.3, 571.4, 571.40, 571.41, 571.42, 571.49, 571,5, 571,6, 571.8, 571.9, 572.3, 572.8, 573.5, V42.0, V42.1, V42.2, V42.3, V42.4, V42.5, V42.6, V42.7, V42.81,V42.82, V42.83, V42.84, V42.89, V42.9, 996.80, 996.81, 996.82, 996.83, 996.84, 996.85, 996.86, 996.87, 996.88, 996.89 |
| Severe cardiac conditions  (chronic heart disease; ccs972: hypertrophic cardiomyopathy, acute/subacute endocarditis, constrictive pericarditis, tamponade; ccs1052: complete AV block, cardiac device insitu; ccs1062: atrial fibrillation, atrial flutter; ccs108: congestive heart failure; cc962: mitral stenosis, atrial stenosis, dual valve disease, value replacement) | 393, 394, 394.0, 394.1, 394.2, 394.9, 395, 395.0, 395.1, 395.2, 395.9, 396, 396.0, 396.1, 396.2, 396.3, 396.8, 396.9, 397, 397.0, 397.1, 397.9, 398, 398.0, 398.9, 398.90, 398.91, 398.99, 414, 414.0, 414.00, 414.01, 414.02, 414.03, 414.04, 414.05, 414.06, 414.07, 414.1, 414.10, 414.11, 414.12, 414.19, 414.2, 414.3, 414.8, 414.9, 416, 416.0, 416.1, 416.8, 416.9, 745, 745.0, 745.10, 745.11, 745.12, 745.19, 745.2, 745.3, 745.4, 745.5, 745.6, 745.60, 745.61, 745.69,745.7, 745.8, 745.9, 746, 746.0, 746.00, 746.01, 746.02, 746.09, 746.1, 746.2, 746.3, 746.4, 746.5, 746.6, 746.7, 746.8, 746.81, 746.82, 746.83, 746.84, 746.85, 746.86, 746.87, 746.89, 746.9, 747.0, 747.1, 747.10, 747.11, 747.2, 747.20, 747.21, 747.22, 747.29, 747.3, 747.4, 747.40, 747.41, 747.42, 747.49, 421.0, 421.1, 421.9, 423.2, 423.3, 425.1, 425.11, 425.18, 426.0, 426.10, V45.0, V45.00, V45.01, V45.02, V45.09, V53.3, V53.31, V53.32, V53.39 427.31, 427.32, 398.91, 428.0, 428.1, 428.20, 428.21, 428.22, 428.23, 428.30, 428.31, 428.32, 428.33, 428.40, 428.41, 428.42, 428.43, 428.9, 394.0, 394.2, 395.0, 395.2, 396.0, 396.1, 396.2, 396.3, 396.8, 396.9, V42.2, V43.3, 421.0, 421.1, 421.9, 423.2, 423.3, 425.1, 425.11, 425.18, 426.0, 426.10, V45.0, V45.00, V45.01, V45.02, V45.09, V53.3, V53.31 V53.32, V53.39, 427.31, 427.32, 398.91, 428.0, 428.1, 428.20, 428.21, 428.22, 428.23, 428.30, 428.31, 428.32, 428.33, 428.40, 428.41, 428.42, 428.43, 428.9, 394.0, 394.2, 395.0, 395.2, 396.0, 396.1, 396.2, 396.3, 396.8, 396.9, V42.2, V43.3 |
| Maternal pregnancy-associated conditions | |
| Pregnancy-induced hypertension | 642.30, 642.32, 642.33, 642.34, 642.40, 642.41, 642.42, 642.43, 642.44, 642.50, 642.51, 642.52, 642.53, 642.54,  642.70, 642.71, 642.72, 642.73, 642.74 |
| Gestational hypertension* | 642.30, 642.32, 642.33, 642.34 |
| Mild preeclampsia/hypertension* | 642.40, 642.41, 642.42, 642.43, 642.44 |
| Severe preeclampsia/hypertension* | 642.50, 642.51, 642.52, 642.53, 642.54 |
| Eclampsia* | 642.6, 642.60, 642.61, 642.62, 642.63, 642.64 |
| Gestational diabetes | 648.8, 648.80, 648.81, 648.82, 648.83, 648.84 |
| Acute Respiratory Distress Syndrome (ARDS)* | 517.3, 518.5, 518.51, 518.52, 518.53, 518.81, 518.82, 518.83, 518.84, 799.1, V46.1, V46.11, V46.12, V46.13, V46.2 |
| Placenta previa* | 641.0, 641.00, 641.01, 641.03, 641.1, 641.10, 641.11, 641.13 |
| Previous Cesarean section* | 596.10, 596.18, 596.20, 596.22, 654.2, 654.20, 654.21, 654.23 |
| Multiple gestation* | 651, 651.00, 651.01, 651.03, 651.10, 651.11, 651.13, 651.20, 651.21, 651.23, 651.30, 651.31, 651.33, 651.40, 651.41, 651.43, 651.50, 651.51, 651.53, 651.60, 651.61, 651.63, 651.70, 651.71, 651.73, 651.80, 651.81, 651.83, 651.90, 651.91, 651.93, V91, V91.00, V91.01, V91.02, V91.03, V91.09, V91.10, V91.11, V91.12, V91.19, V91.20, V91.21, V91.22, V91.29, V91.90, V91.91, V91.92, V91.99 |
| “High-Risk Patients” | Women with one of the following conditions described above: chronic hypertension, pregnancy-induced hypertension (includes gestational, mild preeclampsia/hypertension, severe preeclampsia/hypertension), eclampsia, diabetes mellitus, gestational diabetes, multiple gestation, renal disease, dialysis, organ transplant, placenta previa, severe chronic medical conditions, and severe cardiac conditions |

*Indicates codes used for functional maternal level of care assignment. Gestational age was used to identify necessary subgroups.
