# Supplementary material for: Identifying individual hospital levels of maternal care using administrative data
Source: BMC Health Serv Res. 2021 Jun 2;21:538. doi: 10.1186/s12913-021-06516-y (PMC8171026; doi:10.1186/s12913-021-06516-y)
Supplement: Supplementary file 2 — Additional file 2: Supplemental Table 1. Sensitivity and Specificity of High-Risk Patient Thresholds to Identify Maternal Levels of Care. Supplemental Table 2. Maternal and Infant Characteristics Delivering in Non-Obstetric and Obstetric Hospitals by Empiric Maternal Level of Care in California, 2000–2009. Supplemental Table 3. Maternal and Infant Characteristics Delivering in Non-Obstetric and Obstetric Hospitals by Empiric Maternal Level of Care in Missouri, 2000–2009. Supplemental Table 4. Maternal and Infant Characteristics Delivering in Non-Obstetric and Obstetric Hospitals by Empiric Maternal Level of Care in Pennsylvania, 2000–2009. [file 12913_2021_6516_MOESM2_ESM.docx]

**Title**: Identifying Individual Hospital Levels of Maternal Care using Administrative Data

**Authors**: Sara C. Handley MD, MSCE^1,2^, Molly Passarella MS^1^, Sindhu K. Srinivas, MD, MSCE^2,3^, Scott A. Lorch MD MSCE^1,2^

**Institutions**: ^1^Division of Neonatology, Department of Pediatrics, The Children’s Hospital of Philadelphia and the Perelman School of Medicine-University of Pennsylvania, Philadelphia, PA; ^2^Leonard Davis Institute of Health Economics, University of Pennsylvania, Philadelphia, PA, ^3^The Maternal and Child Health Research Center, Department of Obstetrics and Gynecology, Perelman School of Medicine-University of Pennsylvania, Philadelphia, PA

Supplemental Table 1. Sensitivity and Specificity of High-Risk Patient Thresholds to Identify Maternal Levels of Care

| Maternal Level of Care | High-Risk Patient Type | Empiric optimal cutpoint^a^ | Youden index | Cutpoint sensitivity | Cutpoint specificity | Cutpoint area under ROC curve | Cutpoint same as chosen threshold and rationale | Chosen threshold number of cases/year | Chosen threshold sensitivity | Chosen threshold specificity | Correctly classified with chosen threshold |
| --- | --- | --- | --- | --- | --- | --- | --- | --- | --- | --- | --- |
| Level II | Preterm (<37 week) multiples | 5 | 0.63 | 85% | 77% | 0.81 | No, similar correctly classified^b^ | ≥5 | 88% | 72% | 81% |
|  | Primary cesarean section for placenta previa | 1 | 0.55 | 69% | 86% | 0.78 | No, face validity^c^ | ≥3 | 47% | 96% | 70% |
|  | Severe hypertension after 34 weeks’ gestation | 3 | 0.58 | 71% | 87% | 0.79 | Yes | ≥4 | 71% | 87% | 78% |
| Level III | Previous cesarean section and a placental previa | 1 | 0.39 | 52% | 87% | 0.69 | No, face validity^c^, maximize correct classification | ≥3 | 22% | 96% | 84% |
|  | Preeclampsia before 34 weeks’ gestation | 4 | 0.89 | 93% | 95% | 0.94 | Yes | ≥5 | 93% | 95% | 95% |
|  | Severe hypertension or eclampsia before 34 weeks’ gestation | 2 | 0.91 | 95% | 96% | 0.95 | Yes | ≥3 | 95% | 96% | 96% |
|  | Acute respiratory distress syndrome | 0 | 0.35 | 66% | 69% | 0.67 | No, face validity^c^, maximize correct classification | ≥3 | 19% | 99% | 85% |
| Level IV | Severe chronic medical conditions | 1 | 0.42 | 53% | 90% | 0.71 | No, face validity^c^ | ≥3 | 34% | 100% | 62% |
|  | Severe cardiac conditions | 4 | 0.58 | 84% | 74% | 0.79 | Yes | ≥5 | 84% | 74% | 79% |

^a^Identified by Youden’s index method

^b^The ≥5 vs ≥6 thresholds were very similar for preterm multiples and was the most frequent high-risk patient type to distinguish level I and level II maternal levels of care with an associated correct classification with 0.5%. The study team opted to use the threshold of ≥5 based on an examination of the distribution of preterm multiples in the dataset.

^c^Inadequate face validity was the primary reason the empiric cutpoint identified using Youden’s index was not used as the chosen threshold to identify maternal levels of care at a given hospital. As levels must account for the possibility of an urgent, emergent, or unexpected delivery occurring, thus an empiric cutpoint based on 0 to 2 cases lacked adequate face validity. The detailed sensitivity and specificity output generated was used to inform the chosen threshold in order to maximize correct classification.

Supplemental Table 2. Maternal and Infant Characteristics Delivering in Non-Obstetric and Obstetric Hospitals by Empiric Maternal Level of Care in California, 2000-2009

| **Variable** | **Non-Obstetric** | **Level I** | **Level II** | **Level III** | **Level IV** | **P-value** |
| --- | --- | --- | --- | --- | --- | --- |
| Level | N= 4141 (0.1%) | N= 178951 (3.6%) | N= 2079001 (41.8%) | N= 2055616 (41.3%) | N= 661168 (13.3%) |  |
| Deliveries/year (median, IQR) | 77 (62-89) | 465 (318-581) | 1792 (1292-2284) | 3421 (2873-4153) | 5086 (3836-6627) | <0.001 |
| **Maternal Characteristics, %** (unless otherwise noted) | | | | | | |
| Maternal age (years; median, IQR) | 26 (21-31) | 27 (22-31) | 28 (23-32) | 29 (24-33) | 28 (23-33) | <0.001 |
| Race |  |  |  |  |  | <0.001 |
| White, Non-Hispanic | 2844 (68.68%) | 85802 (47.95%) | 686526 (33.02%) | 674004 (32.79%) | 192432 (29.10%) |  |
| Black, Non-Hispanic | 97 (2.34%) | 5045 (2.82%) | 96197 (4.63%) | 108844 (5.29%) | 57907 (8.76%) |  |
| Hispanic | 896 (21.64%) | 73425 (41.03%) | 1034349 (49.75%) | 947225 (46.08%) | 296376 (44.83%) |  |
| Asian/Pacific Islander | 134 (3.24%) | 11286 (6.31%) | 237182 (11.41%) | 303506 (14.76%) | 105949 (16.02%) |  |
| Other | 170 (4.11%) | 3393 (1.90%) | 24747 (1.19%) | 22037 (1.07%) | 8504 (1.29%) |  |
| Insurance |  |  |  |  |  | <0.001 |
| FFS | 905 (21.85%) | 15474 (8.65%) | 65004 (3.13%) | 55402 (2.70%) | 9715 (1.47%) |  |
| HMO | 630 (15.21%) | 51629 (28.85%) | 939027 (45.17%) | 1090095 (53.03%) | 309440 (46.80%) |  |
| Federal | 2338 (56.46%) | 100247 (56.02%) | 991596 (47.70%) | 849606 (41.33%) | 322418 (48.76%) |  |
| Other | 268 (6.47%) | 11601 (6.48%) | 83374 (4.01%) | 60513 (2.94%) | 19595 (2.96%) |  |
| Education |  |  |  |  |  | <0.001 |
| No High School | 237 (5.72%) | 22084 (12.34%) | 244191 (11.75%) | 188815 (9.19%) | 62019 (9.38%) |  |
| Some High School | 789 (19.05%) | 35855 (20.04%) | 394249 (18.96%) | 345378 (16.80%) | 118114 (17.86%) |  |
| High School Diploma/GED | 1662 (40.14%) | 54718 (30.58%) | 570528 (27.44%) | 503291 (24.48%) | 158971 (24.04%) |  |
| At least Some College | 1372 (33.13%) | 63218 (35.33%) | 836078 (40.22%) | 961975 (46.80%) | 294816 (44.59%) |  |
| Missing | 81 (1.96%) | 3076 (1.72%) | 33955 (1.63%) | 56157 (2.73%) | 27248 (4.12%) |  |
| **Maternal comorbid and pregnancy associated conditions, %** | | | | | | |
| Chronic hypertension | 30 (0.72%) | 865 (0.48%) | 12680 (0.61%) | 15944 (0.78%) | 8464 (1.28%) | <0.001 |
| PIH | 122 (2.95%) | 4055 (2.27%) | 55469 (2.67%) | 65585 (3.19%) | 32203 (4.87%) | <0.001 |
| Severe PIH/Eclampsia | 36 (0.87%) | 811 (0.45%) | 15850 (0.76%) | 21666 (1.05%) | 10999 (1.66%) | <0.001 |
| Gestational diabetes | 190 (4.59%) | 6774 (3.79%) | 106580 (5.13%) | 126053 (6.13%) | 47700 (7.21%) | <0.001 |
| Diabetes mellitus | 22 (0.53%) | 716 (0.40%) | 12648 (0.61%) | 17588 (0.86%) | 8298 (1.26%) | <0.001 |
| Renal disease | 14 (0.34%) | 208 (0.12%) | 2148 (0.10%) | 2190 (0.11%) | 1267 (0.19%) | <0.001 |
| Dialysis | 0 (0.00%) | 3 (0.00%) | 80 (0.00%) | 125 (0.01%) | 99 (0.01%) | <0.001 |
| Organ transplant | 0 (0.00%) | 9 (0.01%) | 98 (0.00%) | 176 (0.01%) | 234 (0.04%) | <0.001 |
| Severe chronic condition^a^ | 2 (0.05%) | 24 (0.01%) | 499 (0.02%) | 754 (0.04%) | 728 (0.11%) | <0.001 |
| Severe cardiac condition^b^ | 3 (0.07%) | 115 (0.06%) | 1835 (0.09%) | 2566 (0.12%) | 1975 (0.30%) | <0.001 |
| Placenta previa | 21 (0.51%) | 652 (0.36%) | 10694 (0.51%) | 14240 (0.69%) | 5198 (0.79%) | <0.001 |
| Placenta previa after CS | 4 (0.10%) | 133 (0.07%) | 2245 (0.11%) | 3468 (0.17%) | 1358 (0.21%) | <0.001 |
| Multiple gestation | 58 (1.40%) | 2696 (1.51%) | 48413 (2.33%) | 69460 (3.38%) | 28473 (4.31%) | <0.001 |
| High-risk patients^c^ | 434 (10.48%) | 15206 (8.50%) | 231364 (11.13%) | 283106 (13.77%) | 116794 (17.66%) | <0.001 |
| Cesarean section | 1092 (26.37%) | 49019 (27.39%) | 601707 (28.94%) | 645060 (31.38%) | 204460 (30.92%) | <0.001 |
| **Neonatal Characteristics, %** (unless otherwise noted) | | | | | | |
| Male | 2096 (50.62%) | 91682 (51.23%) | 1062468 (51.10%) | 1053895 (51.27%) | 338596 (51.21%) | <0.001 |
| Birthweight | 3345 (3005,3657) | 3395 (3090,3714) | 3370 (3051,3685) | 3345 (3007,3660) | 3326 (2975,3660) | <0.001 |
| GA, weeks | 39 (38,40) | 39 (38,40) | 39 (38,40) | 39 (38,40) | 39 (38,40) | <0.001 |
| GA categories, weeks |  |  |  |  |  | <0.001 |
| GA <28 weeks | 29 (0.70%) | 425 (0.24%) | 6353 (0.31%) | 10371 (0.50%) | 5997 (0.91%) |  |
| GA 28-31 weeks | 51 (1.23%) | 931 (0.52%) | 15073 (0.73%) | 24057 (1.17%) | 12136 (1.84%) |  |
| GA 32-36 weeks | 373 (9.01%) | 12554 (7.02%) | 174571 (8.40%) | 200882 (9.77%) | 75432 (11.41%) |  |
| GA 37-41 weeks | 3397 (82.03%) | 152380 (85.15%) | 1758636 (84.59%) | 1705755 (82.98%) | 530503 (80.24%) |  |
| GA >41 weeks | 291 (7.03%) | 12661 (7.08%) | 124368 (5.98%) | 114551 (5.57%) | 37100 (5.61%) |  |

Abbreviations: IQR-interquartile range, FFS-fee for service, HMO-health maintenance organization, GED-general education diploma, PIH-pregnancy induced hypertension, CS-Cesarean section, GA-gestational age

^a^Severe chronic medical conditions: pulmonary hypertension, liver failure, dialysis, and organ transplant

^b^Severe cardiac conditions: chronic heart disease, hypertrophic cardiomyopathy, acute/subacute endocarditis, constrictive pericarditis, tamponade, complete atrioventricular block, cardiac device in situ, atrial fibrillation, atrial flutter, congestive heart failure, mitral stenosis, atrial stenosis, dual valve disease, value replacement

^c^High-risk patients have any of the listed maternal comorbid and pregnancy associated conditions.

Supplemental Table 3. Maternal and Infant Characteristics Delivering in Non-Obstetric and Obstetric Hospitals by Empiric Maternal Level of Care in Missouri, 2000-2009

| **Variable** | **Non-Obstetric** | **Level I** | **Level II** | **Level III** | **Level IV** | **P-value** |
| --- | --- | --- | --- | --- | --- | --- |
| Level | N= 4484 (0.6%) | N= 87528 (12.1%) | N= 268344 (37.0%) | N= 106355 (14.7%) | N= 257647 (35.6%) |  |
| Deliveries/year (median, IQR) | 72 (56,83) | 400 (284,606) | 1032 (783,1384) | 2074 (1901,2263) | 3533 (2578,5954) | <0.001 |
| **Maternal Characteristics, %** (unless otherwise noted) | | | | | | |
| Maternal age (years; median, IQR) | 24 (21,28) | 24 (21,28) | 26 (22,30) | 26 (22,31) | 27 (23,32) | <0.001 |
| Race |  |  |  |  |  | <0.001 |
| White, Non-Hispanic | 4170 (93.00%) | 75719 (86.51%) | 222509 (82.92%) | 73186 (68.81%) | 182049 (70.66%) |  |
| Black, Non-Hispanic | 132 (2.94%) | 6038 (6.90%) | 31083 (11.58%) | 19004 (17.87%) | 53233 (20.66%) |  |
| Hispanic | 93 (2.07%) | 4159 (4.75%) | 8738 (3.26%) | 9867 (9.28%) | 12813 (4.97%) |  |
| Asian/Pacific Islander | 43 (0.96%) | 1005 (1.15%) | 4215 (1.57%) | 2992 (2.81%) | 8102 (3.14%) |  |
| Other | 46 (1.03%) | 607 (0.69%) | 1799 (0.67%) | 1306 (1.23%) | 1450 (0.56%) |  |
| Insurance |  |  |  |  |  | <0.001 |
| FFS | 847 (18.89%) | 17429 (19.91%) | 33720 (12.57%) | 7321 (6.88%) | 13037 (5.06%) |  |
| HMO | 480 (10.70%) | 12168 (13.90%) | 110176 (41.06%) | 47833 (44.97%) | 132188 (51.31%) |  |
| Federal | 2406 (53.66%) | 47942 (54.77%) | 110825 (41.30%) | 46544 (43.76%) | 98757 (38.33%) |  |
| Other | 751 (16.75%) | 9989 (11.41%) | 13623 (5.08%) | 4657 (4.38%) | 13665 (5.30%) |  |
| Education |  |  |  |  |  | <0.001 |
| No High School | 211 (4.71%) | 4185 (4.78%) | 5959 (2.22%) | 4977 (4.68%) | 5600 (2.17%) |  |
| Some High School | 1038 (23.15%) | 20305 (23.20%) | 42344 (15.78%) | 17338 (16.30%) | 32544 (12.63%) |  |
| High School Diploma/GED | 1793 (39.99%) | 34592 (39.52%) | 90025 (33.55%) | 30767 (28.93%) | 69202 (26.86%) |  |
| At least Some College | 1422 (31.71%) | 28158 (32.17%) | 128422 (47.86%) | 50749 (47.72%) | 147636 (57.30%) |  |
| Missing | 20 (0.45%) | 288 (0.33%) | 1594 (0.59%) | 2524 (2.37%) | 2665 (1.03%) |  |
| **Maternal comorbid and pregnancy associated conditions, %** | | | | | | |
| Chronic hypertension | 22 (0.49%) | 671 (0.77%) | 2413 (0.90%) | 1226 (1.15%) | 3878 (1.51%) | <0.001 |
| PIH | 118 (2.63%) | 2889 (3.30%) | 8438 (3.14%) | 4433 (4.17%) | 13342 (5.18%) | <0.001 |
| Severe PIH/Eclampsia | 20 (0.45%) | 457 (0.52%) | 1901 (0.71%) | 1624 (1.53%) | 4675 (1.81%) | <0.001 |
| Gestational diabetes | 106 (2.36%) | 2958 (3.38%) | 9902 (3.69%) | 4932 (4.64%) | 13183 (5.12%) | <0.001 |
| Diabetes mellitus | 8 (0.18%) | 263 (0.30%) | 1291 (0.48%) | 862 (0.81%) | 2800 (1.09%) | <0.001 |
| Renal disease | 12 (0.27%) | 162 (0.19%) | 511 (0.19%) | 230 (0.22%) | 444 (0.17%) | <0.001 |
| Dialysis | 0 (0.00%) | 1 (0.00%) | 2 (0.00%) | 7 (0.01%) | 16 (0.01%) | <0.001 |
| Organ transplant | 0 (0.00%) | 1 (0.00%) | 4 (0.00%) | 10 (0.01%) | 69 (0.03%) | <0.001 |
| Severe chronic condition^a^ | 0 (0.00%) | 9 (0.01%) | 41 (0.02%) | 31 (0.03%) | 166 (0.06%) | <0.001 |
| Severe cardiac condition^b^ | 3 (0.07%) | 67 (0.08%) | 279 (0.10%) | 185 (0.17%) | 654 (0.25%) | <0.001 |
| Placenta previa | 19 (0.42%) | 219 (0.25%) | 949 (0.35%) | 487 (0.46%) | 1447 (0.56%) | <0.001 |
| Placenta previa after CS | 5 (0.11%) | 38 (0.04%) | 177 (0.07%) | 99 (0.09%) | 322 (0.12%) | <0.001 |
| Multiple gestation | 54 (1.20%) | 1288 (1.47%) | 5531 (2.06%) | 3437 (3.23%) | 10858 (4.21%) | <0.001 |
| High-risk patients^c^ | 332 (7.40%) | 8100 (9.25%) | 27343 (10.19%) | 14299 (13.44%) | 41241 (16.01%) | <0.001 |
| Cesarean section | 1266 (28.23%) | 24437 (27.92%) | 73064 (27.23%) | 29495 (27.73%) | 80079 (31.08%) | <0.001 |
| **Neonatal Characteristics, %** (unless otherwise noted) | | | | | | |
| Male | 2272 (50.67%) | 44673 (51.04%) | 137213 (51.13%) | 54327 (51.08%) | 132277 (51.34%) | <0.001 |
| Birthweight | 3317 (2977,3629) | 3345(3005,3657) | 3345(3032,3664) | 3317(2977,3657) | 3317(2948,3657) | <0.001 |
| GA, weeks | 39 (38,40) | 39 (38,40) | 39 (38,40) | 39 (38,40) | 39 (38,40) | <0.001 |
| GA categories, weeks |  |  |  |  |  | <0.001 |
| GA <28 weeks | 8 (0.18%) | 125 (0.14%) | 435 (0.16%) | 616 (0.58%) | 2121 (0.82%) |  |
| GA 28-31 weeks | 16 (0.36%) | 247 (0.28%) | 758 (0.28%) | 1368 (1.29%) | 4344 (1.69%) |  |
| GA 32-36 weeks | 207 (4.62%) | 5079 (5.80%) | 18291 (6.82%) | 10281 (9.67%) | 27270 (10.58%) |  |
| GA 37-41 weeks | 4199 (93.64%) | 81156 (92.72%) | 247305 (92.16%) | 93366 (87.79%) | 222777 (86.47%) |  |
| GA >41 | 54 (1.20%) | 921 (1.05%) | 1555 (0.58%) | 724 (0.68%) | 1135 (0.44%) |  |

Abbreviations: IQR-interquartile range, FFS-fee for service, HMO-health maintenance organization, GED-general education diploma, PIH-pregnancy induced hypertension, CS-Cesarean section, GA-gestational age

^a^Severe chronic medical conditions: pulmonary hypertension, liver failure, dialysis, and organ transplant

^b^Severe cardiac conditions: chronic heart disease, hypertrophic cardiomyopathy, acute/subacute endocarditis, constrictive pericarditis, tamponade, complete atrioventricular block, cardiac device in situ, atrial fibrillation, atrial flutter, congestive heart failure, mitral stenosis, atrial stenosis, dual valve disease, value replacement

^c^High-risk patients have any of the listed maternal comorbid and pregnancy associated conditions.

Supplemental Table 4. Maternal and Infant Characteristics Delivering in Non-Obstetric and Obstetric Hospitals by Empiric Maternal Level of Care in Pennsylvania, 2000-2009

| **Variable** | **Non-Obstetric** | **Level I** | **Level II** | **Level III** | **Level IV** | **P-value** |
| --- | --- | --- | --- | --- | --- | --- |
| Level | N= 2234 (0.2%) | N= 200889 (16.9%) | N= 514368 (43.1%) | N= 280634 (23.5%) | N= 194240 (16.3%) |  |
| Deliveries/year (median, IQR) | 78 (59,85) | 422 (293,543) | 1154 (894,1440) | 2710 (1966,3674) | 3696 (3214,7199) | <0.001 |
| **Maternal Characteristics, %** (unless otherwise noted) | | | | | | |
| Maternal age (years; median, IQR) | 26 (21,31) | 27 (22,31) | 28 (24,33) | 29 (24,33) | 29 (23,33) | <0.001 |
| Race |  |  |  |  |  | <0.001 |
| White, Non-Hispanic | 1929 (86.35%) | 171011 (85.13%) | 397368 (77.25%) | 188507 (67.17%) | 117804 (60.65%) |  |
| Black, Non-Hispanic | 60 (2.69%) | 9210 (4.58%) | 55533 (10.80%) | 42409 (15.11%) | 46876 (24.13%) |  |
| Hispanic | 36 (1.61%) | 8052 (4.01%) | 19322 (3.76%) | 18665 (6.65%) | 6479 (3.34%) |  |
| Asian/Pacific Islander | 18 (0.81%) | 1389 (0.69%) | 8147 (1.58%) | 5917 (2.11%) | 3992 (2.06%) |  |
| Other | 191 (8.55%) | 11227 (5.59%) | 33998 (6.61%) | 25136 (8.96%) | 19089 (9.83%) |  |
| Insurance |  |  |  |  |  | <0.001 |
| FFS | 432 (19.34%) | 42788 (21.30%) | 90094 (17.52%) | 57640 (20.54%) | 33924 (17.46%) |  |
| HMO | 580 (25.96%) | 70956 (35.32%) | 249346 (48.48%) | 132854 (47.34%) | 86325 (44.44%) |  |
| Federal | 1120 (50.13%) | 79044 (39.35%) | 162248 (31.54%) | 85312 (30.40%) | 71843 (36.99%) |  |
| Other | 102 (4.57%) | 8101 (4.03%) | 12680 (2.47%) | 4828 (1.72%) | 2148 (1.11%) |  |
| Education |  |  |  |  |  | <0.001 |
| No High School | 72 (3.22%) | 4212 (2.10%) | 6575 (1.28%) | 4098 (1.46%) | 1903 (0.98%) |  |
| Some High School | 390 (17.46%) | 26547 (13.21%) | 53546 (10.41%) | 30978 (11.04%) | 21398 (11.02%) |  |
| High School Diploma/GED | 909 (40.69%) | 73006 (36.34%) | 152090 (29.57%) | 74289 (26.47%) | 47934 (24.68%) |  |
| At least Some College | 842 (37.69%) | 95523 (47.55%) | 295598 (57.47%) | 167876 (59.82%) | 121040 (62.31%) |  |
| Missing | 21 (0.94%) | 1601 (0.80%) | 6559 (1.28%) | 3393 (1.21%) | 1965 (1.01%) |  |
| **Maternal comorbid and pregnancy associated conditions, %** | | | | | | |
| Chronic hypertension | 14 (0.63%) | 1564 (0.78%) | 4831 (0.94%) | 4068 (1.45%) | 3375 (1.74%) | <0.001 |
| PIH | 116 (5.19%) | 5425 (2.70%) | 15884 (3.09%) | 13671 (4.87%) | 12536 (6.45%) | <0.001 |
| Severe PIH/Eclampsia | 15 (0.67%) | 1101 (0.55%) | 4576 (0.89%) | 4542 (1.62%) | 3832 (1.97%) | <0.001 |
| Gestational diabetes | 63 (2.82%) | 8179 (4.07%) | 21732 (4.22%) | 14966 (5.33%) | 9348 (4.81%) | <0.001 |
| Diabetes mellitus | 5 (0.22%) | 1257 (0.63%) | 3348 (0.65%) | 2427 (0.86%) | 2058 (1.06%) | <0.001 |
| Renal disease | 1 (0.04%) | 353 (0.18%) | 855 (0.17%) | 586 (0.21%) | 540 (0.28%) | <0.001 |
| Dialysis | 0 (0.00%) | 1 (0.00%) | 17 (0.00%) | 17 (0.01%) | 19 (0.01%) | <0.001 |
| Organ transplant | 0 (0.00%) | 11 (0.01%) | 38 (0.01%) | 67 (0.02%) | 65 (0.03%) | <0.001 |
| Severe chronic condition^a^ | 0 (0.00%) | 28 (0.01%) | 120 (0.02%) | 169 (0.06%) | 191 (0.10%) | <0.001 |
| Severe cardiac condition^b^ | 3 (0.13%) | 194 (0.10%) | 742 (0.14%) | 764 (0.27%) | 717 (0.37%) | <0.001 |
| Placenta previa | 7 (0.31%) | 666 (0.33%) | 2368 (0.46%) | 1666 (0.59%) | 1122 (0.58%) | <0.001 |
| Placenta previa after CS | 1 (0.04%) | 115 (0.06%) | 437 (0.08%) | 368 (0.13%) | 253 (0.13%) | <0.001 |
| Multiple gestation | 20 (0.90%) | 1720 (0.86%) | 7495 (1.46%) | 5944 (2.12%) | 4077 (2.10%) | <0.001 |
| High-risk patients^c^ | 216 (9.67%) | 18221 (9.07%) | 52982 (10.30%) | 39654 (14.13%) | 30090 (15.49%) | <0.001 |
| Cesarean section | 792 (35.45%) | 53472 (26.62%) | 144890 (28.17%) | 83256 (29.67%) | 56239 (28.95%) | <0.001 |
| **Neonatal Characteristics, %** (unless otherwise noted) | | | | | | |
| Male | 1166 (52.19%) | 102536 (51.04%) | 263807 (51.29%) | 144018 (51.32%) | 99412 (51.18%) | <0.001 |
| Birthweight | 3289 (2977,3628) | 3373 (3044,3685) | 3373 (3033,3711) | 3345 (2977,3685) | 3316 (2950,3656) | <0.001 |
| GA, weeks | 39(38,40) | 39(38,40) | 39(38,40) | 39(38,40) | 39(38,40) | <0.001 |
| GA categories, weeks |  |  |  |  |  | <0.001 |
| GA <28 weeks | 5 (0.22%) | 249 (0.12%) | 1722 (0.33%) | 2058 (0.73%) | 1514 (0.78%) |  |
| GA 28-31 weeks | 12 (0.54%) | 504 (0.25%) | 3416 (0.66%) | 3808 (1.36%) | 3069 (1.58%) |  |
| GA 32-36 weeks | 187 (8.37%) | 11397 (5.67%) | 36754 (7.15%) | 25717 (9.16%) | 19368 (9.97%) |  |
| GA 37-41 weeks | 2011 (90.02%) | 186974 (93.07%) | 467416 (90.87%) | 247360 (88.14%) | 168674 (86.84%) |  |
| GA >41 weeks | 19 (0.85%) | 1765 (0.88%) | 5060 (0.98%) | 1691 (0.60%) | 1615 (0.83%) |  |

Abbreviations: IQR-interquartile range, FFS-fee for service, HMO-health maintenance organization, GED-general education diploma, PIH-pregnancy induced hypertension, CS-Cesarean section, GA-gestational age

^a^Severe chronic medical conditions: pulmonary hypertension, liver failure, dialysis, and organ transplant

^b^Severe cardiac conditions: chronic heart disease, hypertrophic cardiomyopathy, acute/subacute endocarditis, constrictive pericarditis, tamponade, complete atrioventricular block, cardiac device in situ, atrial fibrillation, atrial flutter, congestive heart failure, mitral stenosis, atrial stenosis, dual valve disease, value replacement

^c^High-risk patients have any of the listed maternal comorbid and pregnancy associated conditions.
